# MicroRNA-3200-3p targeting CAMK2A modulates the proliferation and metastasis of glioma *in vitro*

**DOI:** 10.1080/21655979.2022.2048995

**Published:** 2022-03-14

**Authors:** Haibin Wang, Zhaobin Zeng, Renhui Yi, Jun Luo, Jinming Chen, Jianyun Lou

**Affiliations:** Department of Neurosurgery, The First Affiliated Hospital of Gannan Medical College, Ganzhou, Jiangxi, China

**Keywords:** Glioma, miR-3200-3p, CAMK2A, proliferation, metastasis

## Abstract

MicroRNA (miRNA) is strongly interrelated with the pathogenesis of glioma. However, its potential biological effect and underlying mechanism of miR-3200-3p in human glioma remain elusive. In the current study, we checked the level of miR-3200-3p in different glioma cells. Then, its biological functions on glioma cell proliferation metastasis was investigated using the miR-3200-3p mimic and inhibitor. The direct target of miR-3200-3p was tested in these cells. Results demonstrated that miR-3200-3p is remarkably downregulated in human glioma cells. The relative level of miR-3200-3p is strongly associated with biological features, including proliferation, colony formation, and metastasis. Additionally, Ca^2+^/calmodulin dependent kinase 2a (CAMK2A) might be the direct target gene of miR-3200-3p, and CAMK2A overexpression reversed the anticancer roles of miR-3200-3p on glioma cellular function. Importantly, these results further showed that miR-3200-3p downregulated the proliferation and metastasis by suppressing the expression of CAMK2A, thus regulating the Ras/Raf/MEK/ERK pathway. This study provided provided insights into the biological role of miR-3200-3p, which might function as a potential biomarker in glioma therapy.

## Graphic Abstract

**Figure:**
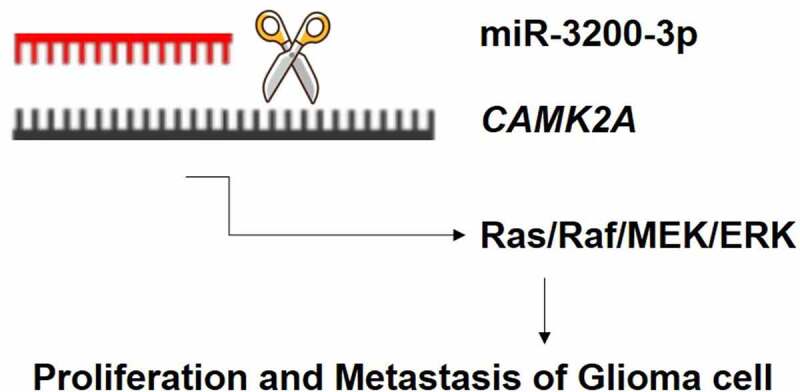

## 1. Introduction

Glioma is a common tumor type in the brain and central nervous system, accounting for 40–50% of the brain tumors. The incidence rate of glioma is high, and its characteristics are low apoptosis and strong invasion [1,2]. To date, chemotherapy and radiotherapy are the main treatment methods. However, considering its special location, difficult diagnosis, complex treatment, and poor prognosis, the average survival rate remains very low, and no clear treatment measure is available [3,4]. The known molecular and cellular mechanisms in glioma pathology are not enough to make significant achievements in targeting and individualization [5]. Therefore, the underlying effects related to the occurrence, development, continuation, and persistence of gliomas should be identified, and the potential mechanisms and regulatory pathways of gliomas should be revealed to provide insights for effectual therapy. More importantly, specific target for glioma diagnosis and prognosis should be determined to improve the survival rate of patients, which might provide more effective or potential treatment for glioma.

In recent years, microRNA (miRNA) biomarkers have brought new ideas for the diagnosis and treatment of glioma and have shown good diagnostic potential. MiRNA is a group of 18–25 bp non-coding RNA sequences, and it interacts with the target gene in the untranslated region of target gene RNA 3 in a sequence-specific mode to inhibit the expression of target gene [6,7]. MiRNAs are involved in different cellular processes. More importantly, they are related to the progress of tumors and are essential for the unlimited proliferation and multi-directional metastasis of various tumor cells [8,9]. MiRNA levels are unbalanced in various malignant cells, such as differentiation, proliferation, metastasis, angiogenesis, and tumorigenesis, including glioma [10]. Many mature miRNAs are exhibited as the prognosis, chemotherapy resistance, and radiotherapy resistance of clinical glioma patients [11]. Several miRNAs can affect the maintenance and development of cancer diseases by regulating the occurrence and development of tumors [12,13]. MiR-3200-3p has the greatest biomarker potential in multiple sclerosis [14]. miR-3200-3p showed a significant downregulated in Alzheimer’s Disease, which is linked with neuronal synaptic functions [15].

Considering that miR-3200-3p is frequently altered in some diseases, the present research hypothesized that it may participate in glioma progression. Therefore, to examine the influences of miR-3200-3p on the developmental and biological behavior of glioma, we detected the expression of miR-3200-3p in several glioma cell lines and measured its biological function in tumor cell proliferation and metastasis. In addition, the current research investigated the relation between miR-3200-3p and Ca^2+^/calmodulin dependent kinase 2a (CAMK2A) and examined their effect on glioma cell progression. We aimed to reveal a novel mechanism of miR-3200-3p in glioma and discussed a potential for its novel use in miR-3200-3p-based therapeutic development.

## 2. Material and method

### 2.1. Cell culture

Human glioma cell lines (U251, T98G and LN229, SHG-44), and one human normal astroglial cell HEB were obtained from ATCC. DMEM supplemented with 10% FBS was utilized to culture cells at 37°C under a 5% CO_2_ environment. Here, the miR-3200-3p mimics, anta-miR-3200-3p, the CAMK2A overexpression plasmid (pcDNA3.1-CAMK2A), and the negative controls were obtained from Shanghai GenePharma Co., Ltd, (Shanghai, China). Approximately 30 nM of miR-3200-3p mimics, anta-miR-3200-3p, or pcDNA3.1-CAMK2A was utilized for cell transfection. In rescue experiments, glioma cells were co-transfected with 30 µM miR-3200-3p mimics together with 10 µg pcDNA3.1-CAMK2A or pcDNA3.1 vector simultaneously. Lipofectamin 2000 (Invitrogen) was utilized for transfection following the manufacturer’s instruction. Approximately 6 h after transfection, cell media were replaced with normal culture media.

### 2.2. Real-time PCR (RT-PCR) assay

RT-PCR assay was performed as reported previously [16]. RNA was prepared from cells, and its purity and concentration were directly examined by NanoDrop. The prepared RNA was immediately reversely transcribed into cDNA. For miR-3200-3p relative expression, RT-PCR was carried out using the miScript SYBR Green PCR kit (Qiagen); while for CAMK2A relative expression, PCR was carried out using the SYBR Green PCR Master Mix kit (Applied Biosystems). All these relative levels have been assayed by the 2^−ΔΔCT^ method. Quantification of miR-3200-3p and miRNA was examined, and their relative levels were controlled to GAPDH and U6, respectively. The following exhibited the primers in the study: hsa-miR-3200-3p forward: 5’- CGCACCTTGCGCTACTCA-3’, hsa-miR-3200-3p reverse: 5’- AGTGCAGGGTCCGAGGTATT-3’, U6 forward: 5’- CTCGCTTCGGCAGCACATATACT-3’, U6 reverse: 5’- ACGCTTCACGAATTTGCGTGTC-3’, CAMK2A forward: 5’- AGTCCAGTTCCAGCGTTCAG-3’, CAMK2A reverse: 5’- CCTGTTTCCGCACTTTGGTG-3’, GAPDH forward: 5’- GTCAAGGCTGAGAACGGGAA-3’, and GAPDH reverse: 5’- AAATGAGCCCCAGCCTTCTC-3’.

### 2.3. Dual-luciferase reporter system

As described by Liu, et al [17], the CAMK2A wild-type (CAMK2A-WT) or mutant (CAMK2A-MUT) was amplified into the psi-CHECKTM2 reporter vector. The construction was co-transfected with miRNA-3200-3p mimics. Then, the relative luciferase activity was measured. The data were obtained and assayed in three dependent experiments.

### 2.4. MTT assay

MTT assay was performed as reported previously [17]. The proliferation in glioma U251 and SHG-44 cells was examined by MTT assay. These glioma U251 cells were cultured with 2 × 10^3^ cells/well. After transfection, cells continued to grow for 1–4 days at 37°C. Subsequently, 10 µg MTT regent was injected into one well and continued to be incubated for 4 h. The supernatant was then discarded and injected with 100 µl DMSO (Sigma-Aldrich, Germany). The result was examined at a 595 nm wavelength.

### 2.5. Colony formation assay

The colony formation assay was performed as reported previously [18]. The transfected cells were subsequently fixed and stained by crystal violet for an additional 1 h. The colonies were counted and imaged by a light microscope (magnification, x200).

### 2.6. Scratch-wound healing assay

As described by Liu et al [17]., cell migrative ability was further detected in glioma U251 and SHG-44 cells. The transfected cells were cultured with FBS-free DMEM into a six-well plate, and then a sterile 200 µl pipette tip was used to scratch a wound field. After 48 h of transfection, the migrated distance of the glioma cells was captured to calculate the migration capability of glioma U251 and SHG-44 cell lines under a light microscope. The relative migrated distance was examined based on the distance of cell migration at 0 h. The experiments were carried out in triplicate, and the mean values were calculated.

### 2.7. Transwell matrigel invasion assay

As described by Liu et al [17]., a transwell chamber (8 um pore size; Corning, NY, USA) was used to calculate U251 and SHG-44 cell invasive ability. A total of 200 µl cells with a density of 2 × 10^5^ was seeded in serum-free DMEM medium and cultured on an upper chamber well with Matrigel (BD Biosciences, NJ, USA). Then, and 500 µl of DMEM supplemented with 10% FBS was filled onto lower chamber wells. Approximately 24 h later, the medium of the upper chamber was discarded. The invasive cells were further fixed for 15 min and stained for additional 10 min. These stained cells were captured in five randomly selected fields.

### 2.8. Protein extraction and western blot assay

As described by Cai et al [18]., the transfected U251 and SHG-44 cells were harvested, washed twice with cold-PBS, and resuspended with lysis buffer (Beyotime, China) with 1 mM PMSF. After 1 h of incubation, the lysed cells were concentrated at 10,000 rpm for 30 min. The extracted protein was examined by NanoDrop, loaded on a 12% SDS-PAGE, and transferred into PVDF membranes (Millipore). The treated membrane was washed with TBST, incubated with skimmed milk (5%) for 60 min, and subsequently incubated with the first antibodies (1:1,000) at 4°C overnight. In another day, these treated membranes were incubated with the secondary antibodies (1:5,000). To detect the protein, we used the enhanced-chemiluminescence detection kit (ECL, Amersham) and examined the protein bands by using ImageJ software. The first antibodies utilized in the study were obtained from cell signaling, including CAMK2A (#3357), Raf (#4432), Ras (#3965), MAPK (Erk1/2) (#9102), phospho-ERK (Thr202/Tyr204, #4370), MEK (#4694), and phospho-MEK (Ser217/221, #3958).

### 2.9. Statistical analysis

The data were analyzed using SPSS, and all the graphs were generated using Graph Pad Prism. Student’s t-test was used to assay two groups, and one-way of variance (ANOVA) was used for the analysis of multiple groups. These data were expressed as mean ± standard deviation (S.D.). Statistical significance was considered at P < .05.

## 3. Results

To examine the influences of miR-3200-3p on the developmental and biological behavior of glioma, we detected the expression of miR-3200-3p in several glioma cell lines and measured its biological function in tumor cell proliferation and metastasis. In addition, the current research investigated the relation between miR-3200-3p and Ca^2+^/calmodulin dependent kinase 2a (CAMK2A) and examined their effect on glioma cell progression. We aimed to reveal a novel mechanism of miR-3200-3p in glioma and discussed a potential for its novel use in miR-3200-3p-based therapeutic development.

### 3.1. miR-3200-3p was downregulated in glioma

**Figure 1.**
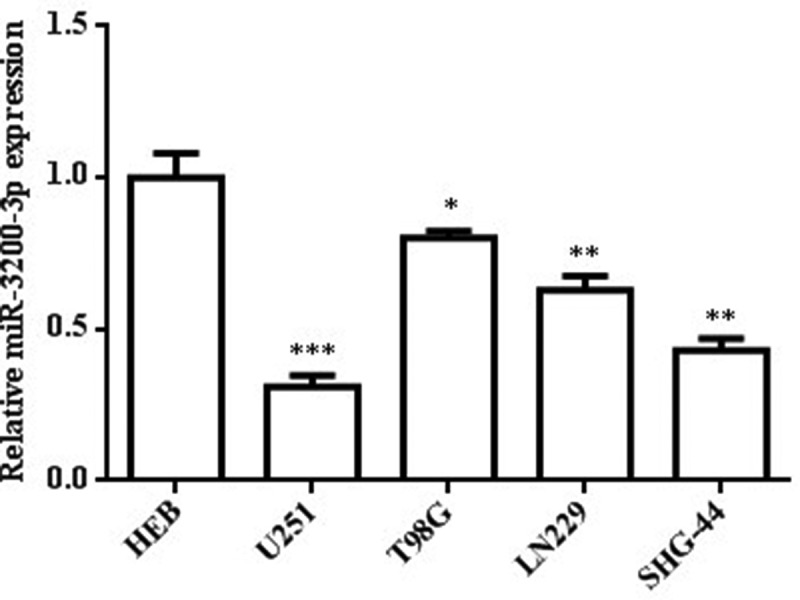
Downregulation of miR-3200-3p in three glioma cells. The level of miR-3200-3p in HEB, U251, T98G, and LN229 was examined by RT-PCR. *p < 0.05, **p < 0.01 and ***p < 0.001 vs. HEB.

To examine the possible relevance of miR-3200-3p in U251 cells, we examined the miR-3200-3p relative level in glioma cell lines. The RT-PCR assay showed that the miR-3200-3p relative level was remarkably downregulated in U251, T98G, and LN229 compared with human normal astroglial cell HEB (Figure 1). Among these glioma cell lines, the U251 cell had the lowest expression (p < 0.001). Thus, the U251 cell was chosen for the following experiment.

### 3.2. miR-3200-3p suppressed the cellular proliferation

**Figure 2.**
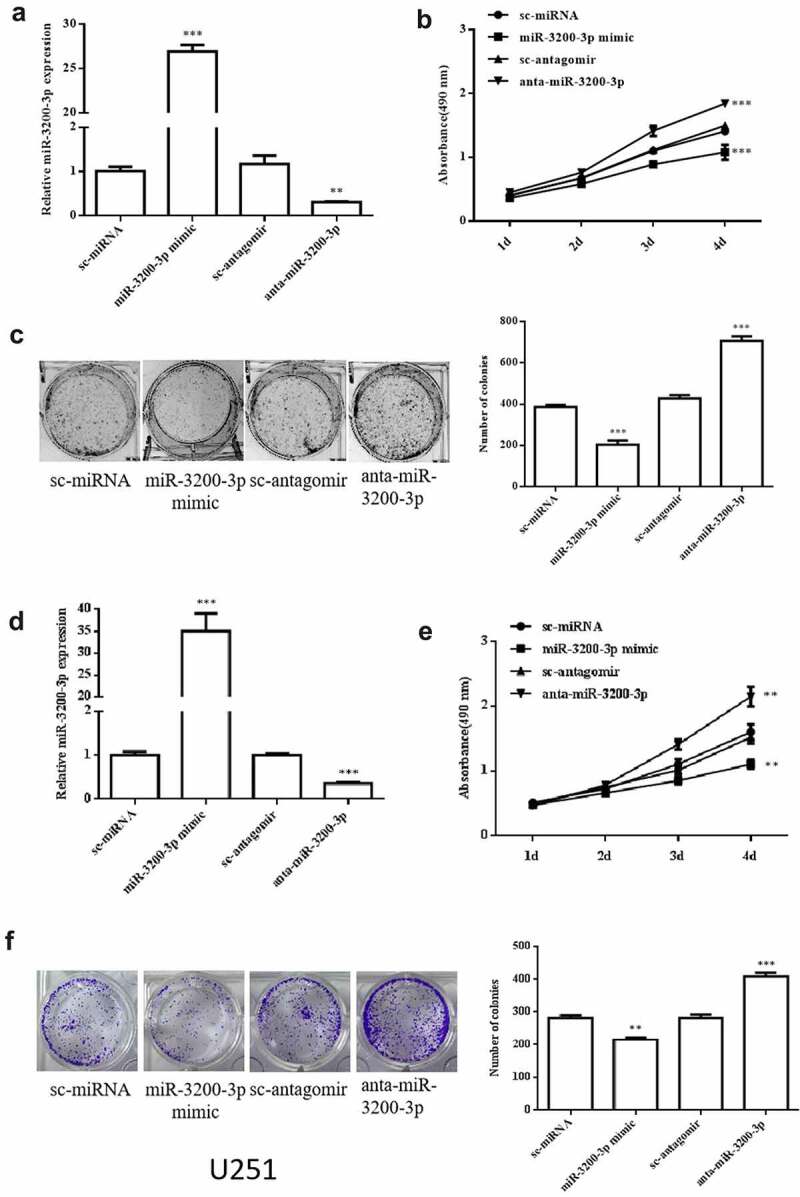
Role of miR-3200-3p on glioma cell proliferation and colony formation. (a) miR-3200-3p expression level in U251 cells was examined by RT-PCR. (b) U251 cell proliferation curves were examined by MTT. (c) U251 cell colony formation. (d) miR-3200-3p expression level in SHG-44 cells. (e,f) SHG-44 cell proliferation curves and colony formation. Representative images (left) and quantitative analysis (right) of colony formation were exhibited. ***p < 0.001 vs. control groups.

To examine whether miR-3200-3p is involved in cellular effect, we transfected the U251 and SHG-44 cells with miR-3200-3p mimic, anta-miR-3200-3p, and the negative controls. The relative level of miR-3200-3p remarkably increased in U251 cells transfected with miR-3200-3p mimic and remarkably decreased after transfected with anta-miR-3200-3p (Figure 2(a)). The MTT assay showed that the cellular proliferation remarkably decreased in the miR-3200-3p mimic group but remarkably increased in anta-miR-3200-3p group in U251 cells at the 4th day (Figure 2(b)). The colony formation indicated that the colony numbers remarkably decreased to 50% compared with the control group, and it was remarkably promoted in the anta-miR-3200-3p group (Figure 2(c)). In SHG-44 cell, cell proliferation and colony formation were deceased by miR-3200-3p mimic (Figure 2(d–f)). Taken together, the data confirmed that miR-3200-3p suppressed cellular proliferation in U251 and SHG-44 cells.

### 3.3. miR-3200-3p inhibited the migration and invasive ability

**Figure 3.**
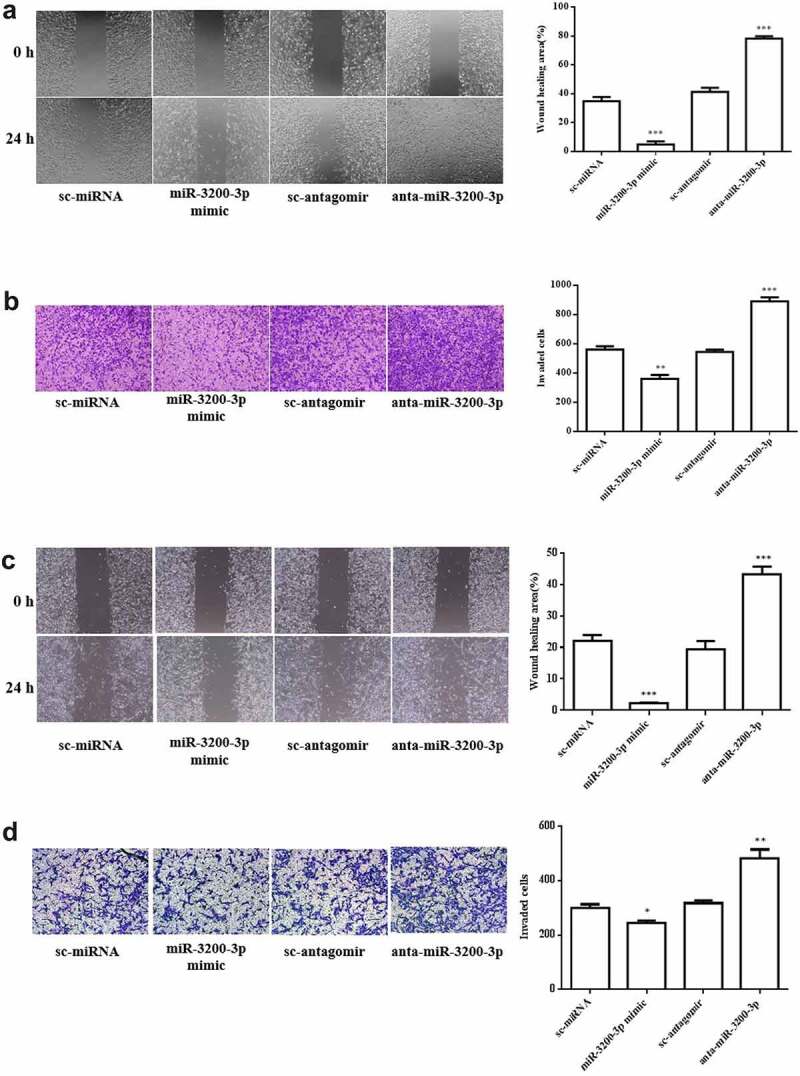
Role of miR-3200-3p on glioma cell metastasis. (a) The U251 cell migratory ability was examined. Representative images (left) showed the migrative cells at 0 and 24 h, and representative quantitative analysis (right) was the relative migration length established from three randomly selected locations. (b) U251 cell invasion was examined by transwell Matrigel invasion assay. (c,d) SHG-44 cell migratory and invasion. Representative images (left) and quantitative analysis (right) of cell invasive ability were exhibited. **p < 0.01 and ***p < 0.001 vs. control groups.

The migration and invasive ability of miR-3200-3p in U251 were further measured. The wound healing assay showed that the wound area in U251 transfected with miR-3200-3p mimic following 24 h was remarkably diminished by 15% compared with NC groups (Figure 3(a)). Subsequently, the transwell Matrigel invasion assay results further indicated that the invasion was remarkably suppressed by 75% compared with the control group (Figure 3(b)). A similar effect was observed in SHG-44 cell after transfection with miR-3200-3p mimic (Figure 3(c,d)). Collectively, the data confirmed that miR-3200-3p resulted in an inhibitory effect on tumor metastasis in U251 and SHG-44 cells.

### 3.4. CAMK2A is a possible gene of miR-3200-3p

**Figure 4.**
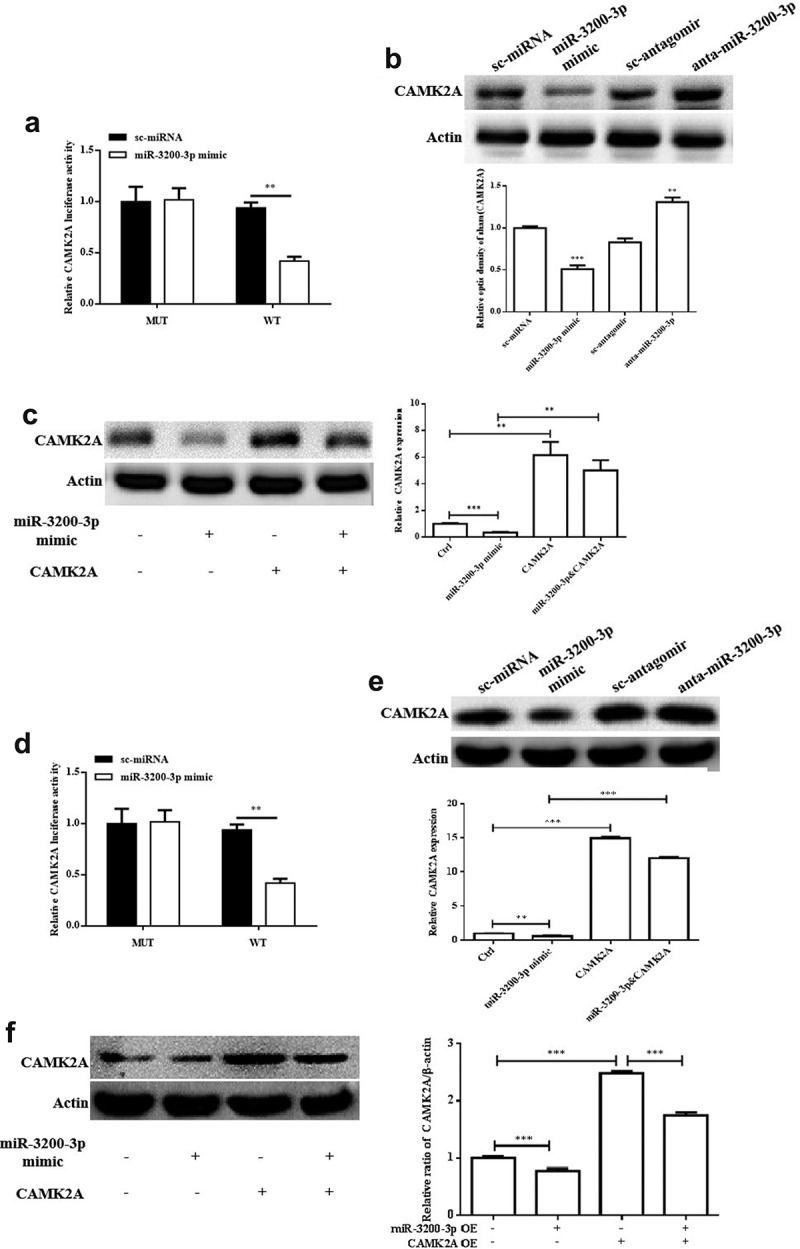
CAMK2A exerts as a direct target of miR-3200-3p. (a) Dual-luciferase activity assay was carried out by using U251 cells. (b) The relative level of CAMK2A was measured by Western blot analysis. (c) The relative level of CAMK2A was examined in glioma U251 cells transfected with or without miR-3200-3p mimic and pcDNA3.1-CAMK2A by Western blot assay. (d) Dual-luciferase activity assay was carried out using SHG-44 cells. (e and f). Relative level of CAMK2A in SHG-44 cells. Representative (upper) and representative (lower) samples showed protein bands and the relative levels, respectively. CAMK2A, Ca^2+^/calmodulin dependent kinase 2a. **p < 0.01 and ***p < 0.001 vs. control groups.

The protein targets of miR-3200-3p were searched using the miRNA target prediction tools miRDB [19,20] and TargetScan [21–23]. A possible target of miR-3200-3p associated with glioma progression is CAMA2A, which is a multifunctional protein kinase. To further confirm the prediction, the U251 cell line was transfected with miR-3200-3p mimic, anta-miR-3200-3p, and the relative controls, and the relative luciferase activity was measured (Figure 4(a)). Western blot analysis was carried out, and the results show that the relative level of CAMK2A could be regulated by miR-3200-3p. The protein relative level of CAMK2A was remarkably downregulated in cells transfected with miR-3200-3p mimics groups, and its expression was remarkably increased by transfecting with miR-3200-3p inhibitor groups (Figure 4(b)). More importantly, the overexpression of CAMK2A remarkably increased the CAMK2A protein expression and have a remarkably reversed effect on the human glioma cells of miR-3200-3p mimics (Figure 4(c)). The pattern of CAMK2A protein level in SHG-44 cells was similar to the result in U251 cell (Figure 4(d–f)). Therefore, CAMK2A could be a considered gene of miR-3200-3p in human glioma.

### 3.5. CAMK2A overexpression reversed the inhibitory role of miR-3200-3p on proliferation

**Figure 5.**
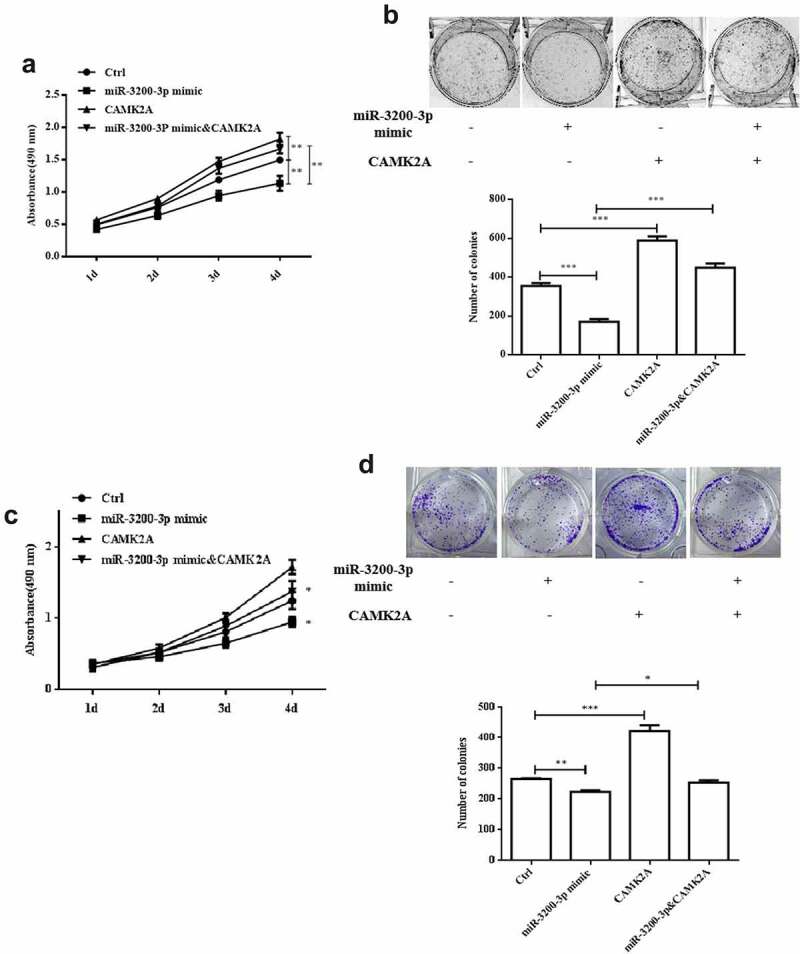
CAMK2A overexpression partially reversed the effect of miR-3200-3p on the ability of proliferation and colony formation. The role of CAMK2A and miR-3200-3p on U251 cell proliferation (a) and the colony formation (b) was examined. The role of CAMK2A and miR-3200-3p on SHG-44 cell proliferation (c) and the colony formation (d) was examined. **p < 0.01 and ***p < 0.001 vs. control groups.

To investigate the interaction between miR-3200-3p and CAMK2A, we transfected the glioma U251 cell with pcDNA3.1-CAMK2A plasmid to rescue the inhibition of CAMK2A induced by miR-3200-3p mimics. As shown in Figure 5, the data showed that miR-3200-3p remarkably diminished the proliferation in U251 cells. By contrast, miR-3200-3p plus CAMK2A recovered the cell growth ability (Figure 5(a)). The colony formation assay indicated that CAMK2A also increased the colony numbers induced by the miR-3200-3p mimics (Figure 5(b)). A similar effect was observed in SHG-44 cell after GAMK2A overexpression with miR-3200-3p mimic (Figure 5(c,d)).

### 3.6. CAMK2A overexpression promoted the migration and invasion induced by miR-3200-3p

**Figure 6.**
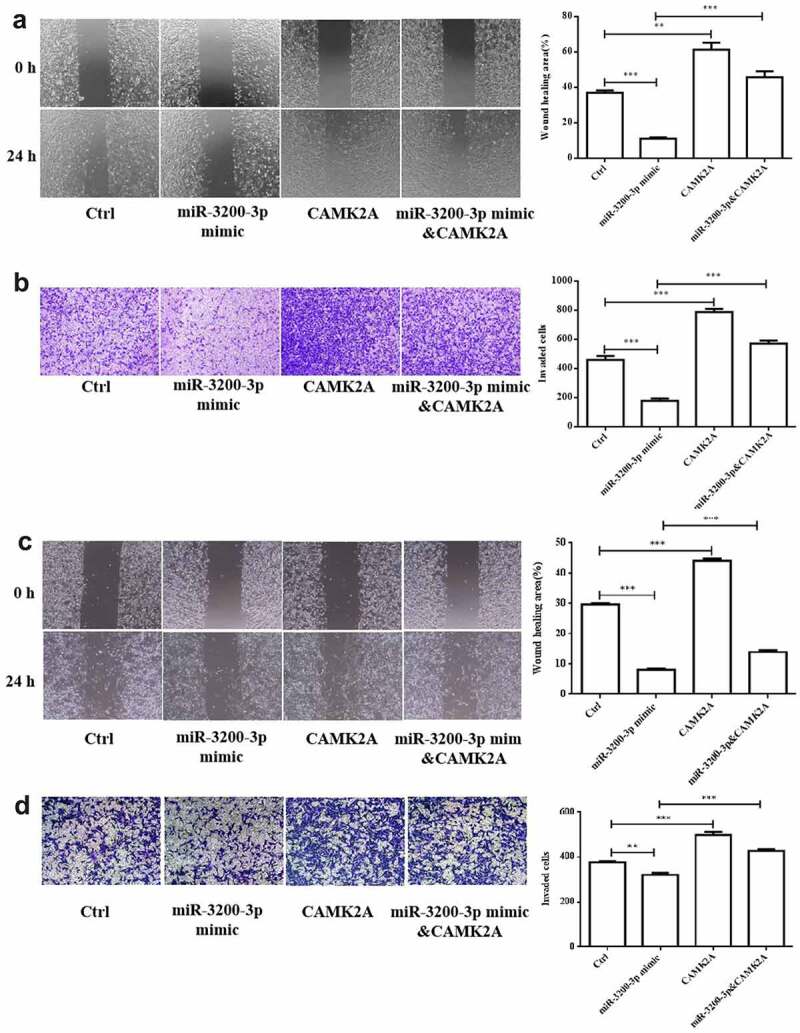
CAMK2A overexpression partially reversed the roles of miR-3200-3p on glioma metastasis. The wound healing (a) and Transwell Matrigel invasion (b) methods were carried out to measure the U251 cell metastasis. (c and d) The same detection in SHG-44 cells. **p < 0.01 and ***p < 0.001 vs. control groups.

Next, the effect of CAMK2A and miR-3200-3p on glioma cell migration and invasion was further investigated. In comparison with the mimic groups, CAMK2A overexpression reversed the wound healing effect of miR-3200-3p (Figure 6(a)). Cell invasion ability was also promoted (Figure 6(b)). The similar phenomenon was observed in SHG-44 cell after GAMK2A overexpression with miR-3200-3p mimic (Figure 6(c,d)). Therefore, CAMK2A overexpression can overcome glioma cell metastasis suppression induced by miR-3200-3p.

### 3.7. miR-3200-3p suppressed the Ras/Raf/MEK/ERK pathway

**Figure 7.**
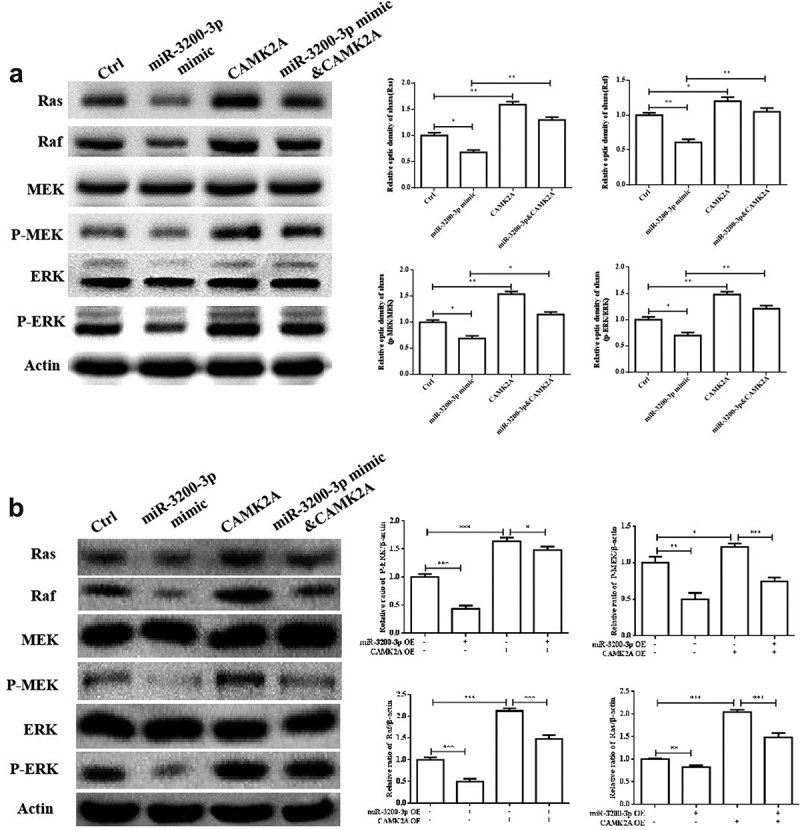
miR-3200-3p suppressed U251 cells via negatively regulating the Ras/Raf/ERK/MEK pathway. (a) The expressions of key elements of the Ras/Raf/ERK/MEK pathway were detected in U251 cells. (b) The relative levels of Ras, Raf, MEK, p-MEK, ERK and p-ERK were tested in U251 cells. (c and d). The same detection in SHG-44 cells. *p < 0.05, **p < 0.01 and ***p < 0.001 vs. control groups.

Considering the role of miR-3200-3p on proliferation and metastasis, some signaling pathways were examined in U251 and SHG-44 cells. The data indicated that miR-3200-3p remarkably downregulated the levels of Ras, Raf, MEK, and ERK (Figure 7), which are important elements of the Raf/Ras/MEK/ERK pathway. Furthermore, the overexpression of CAMK2A reversed this inhibitory effect of miR-3200-3p on the proteins of Raf/Ras/MEK/ERK signaling in glioma cells. Therefore, miR-3200-3p would inhibit the proliferation and metastasis by targeting CAMK2A via the inhibition of Ras/Raf/MEK/ERK pathway.

## 4. Discussion

Glioma is a malignant brain tumor characterized by a high incidence rate, low cure rate, and high mortality. Various treatment schemes can be used for glioma, but no effective treatment method has been found [24,25]. Collectively, further examination is needed to explain the potential mechanism and treatment of glioma. Therefore, new molecular markers should be identified to predict the clinical manifestations and therapeutic effects of glioma. MiRNAs are important regulatory molecules, which exhibit an abnormal expression in gliomas. miRNA-221 and miRNA-222 showed a remarkably increasing expression in human glioma tissues [26]. miRNA generally targets mRNA degradation or delayed translation to regulate different target genes, thus playing a biological mechanism. For example, miRNA-182-5p negatively modulated protocadherin 8 and affected the proliferation and invasion in glioma [27]. miRNA-323-5p regulated insulin-like growth factor 1 receptor (IGF-1 R) and suppressed human glioma cellular growth and enhanced apoptosis [28]. miRNAs not only regulate cell apoptosis and metastasis, but also related to tumor development.

In the current study, our data presented that miR-3200-3p level was downregulated in at least two type of glioma cells, including U251 and SHG-44. Thus, we investigated the biological effects of miRNA-3200-3p in both glioma cells lines for the first time. Results show that miRNA-3200-3p overexpression remarkably inhibited proliferation and colony formation compared with NC groups. The increased miRNA-3200-3p remarkably limited the tumor metastasis in glioma cells. All above results in both glioma cells are consistent. Therefore, miRNA-3200-3p played an important progress in the glioma tumorigenesis.

Subsequently, the luciferase reporter system revealed that CAMK2A might be the direct target gene of miRNA-3200-3p. Furthermore, the overexpression or downregulation of miRNA-3200-3p significantly increased or decreased CAMK2A expression, respectively. CAMK2A is an isoform of CMAK family and is highly expressed in the brain. It is a critical calcium signaling molecule that mediates the Ca^2+^-induced signaling, thus regulating multiple cell functions, including proliferation, migration, and invasion [29,30]. The gain-of-function studies confirmed that CAMK2A overexpression remarkably suppressed glioma cell proliferation and metastasis induced by miR-3200-3p. This finding confirmed that miR-3200-3p is a potential tumor inhibitor by directly targeting CAMK2A.

In addition, the molecular mechanisms of proliferation and metastasis inhibition induced by miR-3200-3p were further investigated. Here, our results examined that the relative levels of Ras, Raf, MEK, ERK, and corresponding phosphorylated molecules were downregulated in miR-3200-3p mimic glioma cells. Importantly, CAMK2A overexpression would promote the major molecules of the Ras/Raf/MEK/ERK and reverse the inhibitory roles induced by miR-3200-3p mimic. Therefore, miR-3200-3p may play critical roles in suppressing glioma cell proliferation and metastasis via downregulating multiple target factors in the Ras/Raf/MEK/ERK pathway.

## 5. Conclusion

In summary, we provided the first evidence that the miR-3200-3p relative level was remarkably downregulated in human glioma cell *in vitro*. The miR-3200-3p overexpression suppressed the proliferation, migration, and invasion via targeting CAMK2A expression, thus regulating the Ras/Raf/MEK/ERK pathway. Collectively, our findings on miR-3200-3p are encouraging and suggest that exogenous downregulation of miR-3200-3p could be a possible approach for glioma therapy in future.

## Data Availability

All the data were in the manuscript.
